# Estimating the Effect of Early Treatment Initiation in Parkinson's Disease Using Observational Data

**DOI:** 10.1002/mds.28339

**Published:** 2020-10-27

**Authors:** Lieneke van den Heuvel, Luc J.W. Evers, Marjan J. Meinders, Bart Post, Anne M. Stiggelbout, Tom M. Heskes, Bastiaan R. Bloem, Jesse H. Krijthe

**Affiliations:** ^1^ Center of Expertise for Parkinson & Movement Disorders, department of Neurology, Donders Institute for Brain, Cognition and Behaviour Radboud University Medical Center Nijmegen the Netherlands; ^2^ Institute for Computing and Information Sciences Radboud University Nijmegen the Netherlands; ^3^ Scientific Center for Quality of Healthcare (IQ Healthcare), Radboud Institute for Health Sciences Radboud University Medical Center Nijmegen the Netherlands; ^4^ Department of Biomedical Data Sciences, Medical Decision Making Leiden University Medical Centre Leiden the Netherlands; ^5^ Department of Intelligent Systems Delft University of Technology Delft the Netherlands

**Keywords:** observational data, Parkinson's disease, PPMI treatment initiation

## Abstract

**Background:**

Both patients and physicians may choose to delay initiation of dopamine replacement therapy in Parkinson's disease (PD) for various reasons. We used observational data to estimate the effect of earlier treatment in PD. Observational data offer a valuable source of evidence, complementary to controlled trials.

**Method:**

We studied the Parkinson's Progression Markers Initiative cohort of patients with de novo PD to estimate the effects of duration of PD treatment during the first 2 years of follow‐up, exploiting natural interindividual variation in the time to start first treatment. We estimated the Movement Disorder Society–Unified Parkinson's Disease Rating Scale (MDS‐UPDRS) Part III (primary outcome) and several functionally relevant outcomes at 2, 3, and 4 years after baseline. To adjust for time‐varying confounding, we used marginal structural models with inverse probability of treatment weighting and the parametric g‐formula.

**Results:**

We included 302 patients from the Parkinson's Progression Markers Initiative cohort. There was a small improvement in MDS‐UPDRS Part III scores after 2 years of follow‐up for patients who started treatment earlier, and similar, but nonstatistically significant, differences in subsequent years. We found no statistically significant differences in most secondary outcomes, including the presence of motor fluctuations, nonmotor symptoms, MDS‐UPDRS Part II scores, and the Schwab and England Activities of Daily Living Scale.

**Conclusion:**

Earlier treatment initiation does not lead to worse MDS‐UPDRS motor scores and may offer small improvements. These findings, based on observational data, are in line with earlier findings from clinical trials. Observational data, when combined with appropriate causal methods, are a valuable source of additional evidence to support real‐world clinical decisions. © 2020 The Authors. *Movement Disorders* published by Wiley Periodicals LLC on behalf of International Parkinson and Movement Disorder Society

First‐choice treatments in persons with Parkinson's disease (PD) focus on pharmacological dopamine replacement, which suppresses symptoms and improves quality of life.^1^, ^2^ However, both patients and physicians may choose to delay initiation of dopaminergic treatment for various reasons, including concerns that treatment might negatively affect disease progression or that early treatment initiation might promote the earlier onset of side effects. Although evidence that counters these concerns is growing, the influence of dopaminergic treatment on long‐term disease progression is not fully elucidated.^3^, ^4^, ^5^

Several studies, including randomized controlled trials (RCTs), explored the effect of dopaminergic treatment on disease progression in patients with de novo PD using clinical, pathological, or biomarker end points. Three large studies evaluated levodopa (ELLDOPA study,^2^ PD‐MED study,^6^ and LEAP study^1^), but none found clinical evidence that levodopa accelerates progression of PD, and only the ELLDOPA and PD‐MED studies reported evidence for potential long‐term benefits of levodopa treatment. Other studies examined the effect of monoamine oxidase B (MAO‐B) inhibitors. A possible disease‐modifying effect was found in animal models of parkinsonism, but subsequent RCTs in humans (which tested rasagiline or selegiline) could not confirm this.^7^, ^8^ Similarly, there is insufficient evidence that the use of dopamine agonists delays disease progression.^9^

Although RCTs remain essential as the most valid source of evidence to establish treatment effects, controlled trials have limitations, including a relatively short follow‐up (in the aforementioned studies typically <2 years, except for the PD‐MED study with a 3‐year follow‐up) or having selected populations with strict inclusion and exclusion criteria (eg, exclusion of patients with severe tremor in the ELLDOPA and LEAP studies). Also, interventions delivered in trial settings may not always correspond to real‐world prescription practices because the choice to start treatment in real life is complex, including many different aspects such as the physicians' preferences and experience (eg, influenced by their own training or how they balance expected symptomatic benefits vs. side effects) and patients' preferences. Recognition is therefore growing that findings of strictly controlled trials cannot be extrapolated automatically to decisions for real‐life patients, particularly for disorders with heterogeneous and variable presentations.^10^, ^11^

Observational studies offer potential advantages, including a more representative patient population and larger samples with longer follow‐up.^12^ Analyzing observational data comes with its own challenges, such as the necessity for additional causal and statistical assumptions to adequately adjust for confounding (eg, time‐varying confounding in which the values of potential confounders may change over time in response to treatment decisions). When such challenges in analyzing observational data are addressed carefully, these types of data can be valuable sources of complimentary evidence regarding the outcomes of real‐world clinical decisions.

Our goal here is to estimate the long‐term effect of early or delayed start of treatment in patients with de novo PD using longitudinal, observational data. To correct for confounding we applied 4 different methods, including 2 models that not only correct for baseline confounding but also consider the time‐varying nature of confounding, that is, an inverse probability of treatment weighting model and adjustment using the parametric g‐formula.^13^

## 1 Methods

### 1.1 Study Design

We used observational data from a de novo PD cohort, the Parkinson's Progression Markers Initiative (PPMI), to estimate the effects of early initiation of PD treatment. Specifically, we estimated the effect of duration of PD treatment in the first 2 years of follow‐up, exploiting the fact that—dictated by preferences of professionals and patients, differences in loco‐regional treatment protocols, and other factors—there would be natural interindividual variation in the time when the first treatment since diagnosis is initiated. This natural variation in observational data resembles a “delayed start” design, albeit not randomized. We were particularly interested to see if the disease course (ie, outcomes at 2, 3, and 4 years of follow‐up) would be different for early starters versus later starters after correction for confounding factors that might have influenced both the decisions to initiate treatment at each time point and the outcome measures. Consequently, adjusting for differences at baseline between groups of patients who had different durations of PD medication use is insufficient^14^; it is also necessary to consider time‐varying confounding caused by treatment decisions based on the patient status at each point during the disease. Therefore, we compared 4 different methods to address confounding, including a naive model that directly compares groups of patients with different durations of treatment during the first 2 years of follow‐up, a linear model correcting for baseline variables, inverse probability of treatment weighting, and adjustment using the parametric g‐formula.^13^

### 1.2 PPMI Cohort

PPMI is an observational, longitudinal, multicenter study designed to establish clinical, imaging, and biosample data to define biomarkers of PD progression. The PPMI cohort used here contained data from 423 recently diagnosed patients.^15^ Broad inclusion criteria included the following: (1) presence of asymmetric resting tremor, asymmetric bradykinesia, or 2 of bradykinesia, resting tremor, and rigidity; (2) diagnosis of PD for <2 years and not taking PD medications at screening; and (3) deficit consistent with PD on single‐photon emission computed tomography imaging. Patients were expected not to require PD medication within at least 6 months from baseline, but were retained in the study and continued treatment if this was required anyway. Data were accessed on May 31, 2019 (www.ppmi-info.org). Each participating PPMI site received ethical approval before study initiation and obtained written informed consent from all participants.

For our analyses, we used data from baseline until 4 years of follow‐up because after that many participants dropped out. In addition to the scheduled biannual study visits, we considered special symptomatic treatment visits: if the participant was willing, an additional visit was planned right before initiation of medication therapy. If this visit was within 3 months of the next planned visit, it replaced the planned visit. In that case, we also treated the symptomatic treatment visit as the planned visit in our analyses.

### 1.3 Duration of PD Treatment

To determine the duration of PD treatment during the first 2 years of follow‐up in PPMI, we considered the number of biannual visits where the patient was registered as using PD medication. We excluded 7 patients who discontinued using PD medication during study follow‐up. We used a linear marginal structural model for the time on treatment. To avoid a mismatch between the continuous time treatment information and the discrete time confounding adjustments in our models, we defined the time on treatment as the number of treatment periods the patient is recorded to be using PD medication multiplied by the length of the time periods (0.5 year). This assumes that a patient used medication during the entire 6‐month period preceding the visit at which they were first recorded to be taking medication. This corresponds to the idea that medication decisions likely took place during the previous visit and that this decision was based on the patient's status during that visit. This allowed us to adjust for the fact that patient status influences medication decisions (see the Statistics section). For further details and information on imputation of missing treatment status, please see Appendix S1. In the main analysis, we included any type of PD medication. In addition, we separately analyzed the effect of the duration of treatment with levodopa only.

### 1.4 Outcomes

The primary outcome was the total score on the MDS‐UPDRS Part III in the *off* state measured at 2, 3, and 4 years after the start of follow‐up. We selected the annual study visits because these included assessments in the *off* state at least 6 hours after the last intake of levodopa/dopamine agonist. Secondary outcomes included the Modified Schwab and England Activities of Daily Living (MSE‐ADL) Scale, presence of dyskinesias and motor fluctuations (based on items 4.1 and 4.3 of the MDS‐UPDRS Part IV), the Questionnaire for Impulsive‐Compulsive Disorders Current Short, MDS‐UPDRS Part I, MDS‐UPDRS Part II, Montreal Cognitive Assessment, and Scales for Outcomes in PD–Autonomic Dysfunction. Because the efficiency of the estimators of some of our models improved by having access to complete observations at all time points under consideration, we imputed missing values (procedure described in Appendix S1).

By using *off* state measurements, we minimized the (dose‐dependent) influence of short‐term effects of dopaminergic treatment on the MDS‐UPDRS Part III. However, dopaminergic medication also has a long‐duration response, which has not yet worn off after 6 hours. This long‐duration response was not present in all patients when the outcome measures were collected because some patients had not started treatment at years 2, 3, and 4. To mitigate this bias, we only considered the subpopulation of patients who had started treatment at the time of the assessments. We should note that this introduces a different problem because it amounts to estimating the causal effect on a subpopulation (only starters) that differs from the whole PPMI population. Therefore, we present additional analyses in Appendix S1 where we extrapolated the treatment effects to the whole sample of 416 patients (excluding only the 7 patients who stopped medication therapy) by adjusting for missing measurements and for missing outcomes for those who had not yet started therapy.

### 1.5 Statistics

Our goal was to estimate the effects of variations in duration of PD treatment on disease‐related outcomes. We did this by considering a treatment policy where every patient is treated for the same amount of time and estimating the effect of changing the treatment duration in this policy. For example, we aimed to answer the question “if all patients would receive 0.5 year treatment within the first 2 years of follow‐up, what would the average change in outcomes be if we changed this to 1.5 years?” We present the estimated linear effect of a change in treatment duration of 1 year.

To make these estimates, we expected that it would be insufficient to adjust merely for differences at baseline between groups of patients who had different durations of PD treatment.^14^

We used 2 methods to adjust for time‐varying confounding: marginal structural models using inverse probability of treatment weighting (IPTW)^16^, ^17^ and the parametric g‐formula.^18^, ^19^, ^20^ For both approaches, we include the following covariates in our models: MDS‐UPDRS Parts I, II, and III scores; MSE‐ADL scores; Montreal Cognitive Assessment scores; age; sex; years of education; presence of cardiovascular disease; and disease duration. The IPTW uses models for the probability of initiating treatment at each visit to correct for time‐varying confounding. To estimate these probabilities, we used linear logistic regression models on the measurements of the previous visit, estimated without pooling, and we used stabilized weights in the marginal structural model. The parametric g‐formula requires a model for disease progression over time, for which we used linear regression models estimated at each time point, without pooling. We expect progression to be more difficult to model accurately than the treatment decision, but it may lead to smaller uncertainty in the estimates. The reason for considering both approaches is that if both methods agree on an estimate, it is less likely that the results are merely caused by an improper model for treatment initiation or disease progression. A detailed description of both approaches and the underlying causal model are given in Appendix S1.

To demonstrate the effect of adjusting for time‐varying confounding, we also fitted a naive model that directly compared patient groups with different durations of treatment during the first 2 years of follow‐up. We extended this naive model to a linear model that adjusts for the value of the outcome at baseline, and we further extended this second model by using inverse probability weighting to correct only for missingness.

In addition to these main analyses, we conducted various additional analyses (see Appendix S1). For example, we include the results of models that also adjust for levodopa equivalent daily dose (LEDD) at the time of collection of the outcomes (Figs. S2 and S4 in Appendix S1), and we estimated the nonlinear effect of starting therapy at the different times (Figs. S8 and S9 in Appendix S1). For the IPTW methods, we estimated interactions between the effect of treatment duration and baseline covariates to consider whether different subgroups of patients respond differently to early treatment initiation (Fig. S10 in Appendix S1). To gauge the sensitivity of our estimates to choices in the probability of treatment models, we also considered models that consider the full history of measurements as well as nonparametric models (Fig. S13 in Appendix S1). In addition, we estimated the effect using a doubly robust targeted maximum likelihood estimator (Fig. S14 in Appendix S1).^21^

For all models, parameter uncertainty was estimated using bootstrapping, subsampling the patients, with 1000 repeats. The reported intervals are ±2 times the standard deviation of the bootstrap distribution of the parameters. All analyses were performed using R 3.6.3^22^; the code to reproduce the analyses is available at https://www.github.com/jkrijthe/pdmed.

## 2 Results

**FIG. 1:**
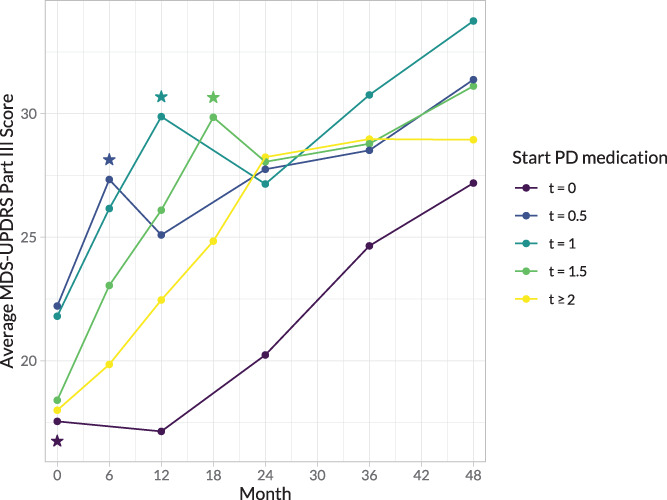
Average MDS‐UPDRS Part III in the *off* state scores in the first 4 years, grouped by the assumed time point when treatment with PD medication was started. Scores are obtained during biannual measurements until treatment initiation and annual measurements after treatment initiation. Star indicates when this group started PD medication. MDS‐UPDRS, Movement Disorder Society–Unified Parkinson's Disease Rating Scale; PD, Parkinson's disease. [Color figure can be viewed at wileyonlinelibrary.com]

From the initial 423 PPMI patients, 7 who stopped using PD medication during follow‐up and 56 whose data about treatment start were missing were excluded from the main analyses. The number of patients available for the analyses at years 2 (n = 302), 3 (n = 311), and 4 (n = 295) varied because we also excluded patients who had not started treatment at the time when the outcome measures were collected, and we excluded patients who dropped out. For the MDS‐UPDRS III, fewer measurements were available because not all assessments fulfilled the *off* criteria and were therefore considered missing; 155 MDS‐UPDRS III measurements were available at year 2, 178 at year 3, and 194 at year 4. Table 1 presents the baseline demographics and disease characteristics of the patients who were registered as using PD medication at year 2 of follow‐up. These patients used levodopa (45%), dopamine agonists (16%), others (13%), or a combination (25%) (Table S2 in Appendix S1). The “others” category included mainly MAO‐B inhibitors. The purely descriptive, average MDS‐UPDRS III scores (*off* state) over time, for the different PD medication duration groups, are presented in Figure 1.

**TABLE 1:** Demographics and disease characteristics of patients (n = 302) included in the main analyses at year 2. Values at baseline are presented, unless stated otherwise.

Variable	Value
Age, y, mean (SD)	61.3 (9.7)
Sex, men, n (%)	202 (67)
Education, y, mean (SD)	15.6 (2.9)
Disease duration, y, mean (SD)	0.5 (0.5)
MDS‐UPDRS Part I, mean (SD)	5.6 (4.1)
MDS‐UPDRS Part II, mean (SD)	6.2 (4.2)
MDS‐UPDRS Part III, mean (SD)	21.1 (8.8)
MoCA, mean (SD)	27.1 (2.3)
MSE‐ADL, mean (SD)	92.8 (6.0)
QUIP‐S, n (%) with a positive rating	57 (19)
SCOPA‐AUT, mean (SD)	9.7 (5.9), missing: 1
Duration of PD medication use within the first 2 years of follow‐up, n (%)	
Start treatment at t = 0 y	31 (10)
Start treatment at t = 0.5 y	180 (60)
Start treatment at t = 1 y	54 (18)
Start treatment at t = 1.5 y	37 (12)
LEDD at 2 y, mean (SD)	370 (292.7)
LEDD at 3 y, mean (SD)	469.9 (330.4), missing: 19
LEDD at 4 y, mean (SD)	546.0 (349.3), missing: 42

MDS‐UPDRS, Movement Disorders Society–Unified Parkinson Disease Rating Scale; MoCA, Montreal Cognitive Assessment Scale; MSE‐ADL, Modified Schwab and England Activities of Daily Living; QUIP‐S, Questionnaire for Impulsive‐Compulsive Disorders in Parkinson's Disease Current Short; SCOPA‐AUT, Scales for Outcomes in PD–Autonomic Dysfunction; PD, Parkinson's disease; LEDD, levodopa equivalent daily dose.

**FIG. 2:**
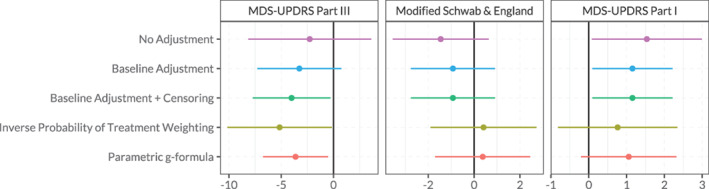
Effect of an additional year of PD treatment on outcomes after 2 years in the subpopulation of patients that had started therapy at the time of measurement, estimated using different methods. For the MDS‐UPDRS, higher scores correspond to worse outcomes, for the Modified Schwab and England, lower scores correspond to worse outcomes. MDS‐UPDRS, Movement Disorder Society–Unified Parkinson's Disease Rating Scale. [Color figure can be viewed at wileyonlinelibrary.com]

The importance of adjusting for time‐varying confounding for answering causal questions about dynamic treatment decisions becomes apparent in Figure 2. When time‐varying confounding was not considered, negative outcomes are attributed to treatment rather than to the worse disease state at the time of treatment initiation. This may be explained by the fact that patients in a worse disease state warrant treatment initiation earlier compared with patients with relatively mild disease stages. Models that consider the time‐varying nature of treatment initiation correct for this confounding effect. Specifically, Figure 2 shows that, when adjusting for time‐varying confounding, the estimated effect of longer PD treatment duration on MDS‐UPDRS III scores was slightly more beneficial compared with models that do not control for confounding or only control for baseline confounding. The influence of controlling for confounding is also seen in our secondary outcomes. For example, the point estimates of the treatment effects on the MSE‐ADL Scale shift from negative to close to zero, and on the MDS‐UPDRS I from a significantly negative to a less negative effect where confidence intervals include zero. Results from all models for all outcome measures are presented in Appendix S1 (Figs. S2–S7).

**FIG. 3:**
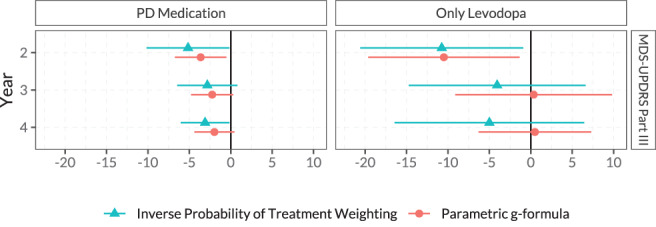
Effect of an additional year of PD treatment (left, any PD medication; right, only levodopa) during the first 2 years of follow‐up in the Parkinson's Progression Markers Initiative. Effect on MDS‐UPDRS Part III *off* score measured at years 2, 3, and 4 for inverse probability of treatment weighting and the parametric g‐formula. Only patients who had started medication treatment at the time of the outcome measurement are included in each analysis. MDS‐UPDRS, Movement Disorder Society–Unified Parkinson's Disease Rating Scale; PD, Parkinson's disease. [Color figure can be viewed at wileyonlinelibrary.com]

**FIG. 4:**
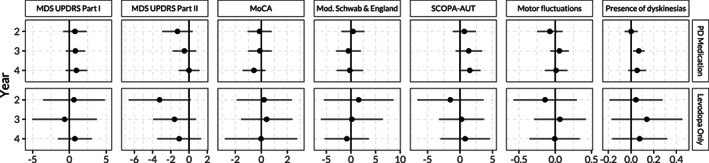
Effect of an additional year of PD treatment (any PD medication) during the first 2 years of follow‐up in the Parkinson's Progression Markers Initiative. Effect on auxiliary outcomes at years 2, 3, and 4 for inverse probability of treatment weighting. Only patients who had started medication treatment at the time of the outcome measurement are included in the analysis. MDS‐UPDRS, Movement Disorder Society–Unified Parkinson's Disease Rating Scale; MoCA, Montreal Cognitive Assessment Scale; Mod., modified; PD, Parkinson's disease; SCOPA‐AUT, Scales for Outcomes in PD–Autonomic Dysfunction.

Figure 3 shows the estimated treatment effects of an additional year of PD medication on the MDS‐UPDRS III scores at 2, 3, and 4 years of follow‐up. The point estimates of the treatment effect indicated an improvement of MDS‐UPDRS III scores at years 2, 3, and 4 of follow‐up, although most 95% confidence intervals included zero. The point estimates of the effects on MDS‐UPDRS I and Scales for Outcomes in PD–Autonomic Dysfunction scores indicated a small worsening effect, but all confidence intervals included zero (Fig. 4 and Figs. S2–S7 in Appendix S1). We found no effect on other secondary outcomes except for an increased presence of dyskinesias at year 3. However, the confidence intervals at years 2 and 4 both included zero. Similar results emerged from the analysis extrapolating the estimated treatment effect for the subpopulation of only starters to the whole PPMI population (Figs. S3 and S6 in Appendix S1).

Analyzing the effects of 1‐year longer treatment with exclusively levodopa (and no other PD medication) (n = 82) showed an improvement of MDS‐UPDRS III scores at year 2, but nonsignificant effects at years 3 and 4 (Fig. 3) with wide confidence intervals. The effects on the other outcomes were also inconclusive with wide confidence intervals (Fig. 4).

## 3 Discussion

The debate about whether dopaminergic pharmacotherapy for PD is merely symptomatic or perhaps also affecting the rate of disease progression is not fully resolved. Prior well‐controlled RCTs found no evidence for a disease‐modifying effect, but these studies may not necessarily have reflected real‐life prescription behavior, which could result in other outcomes. Here we describe the long‐term effects of variations in medication prescription behavior (specifically, differences in time to start of first pharmacotherapy in patients with de novo PD) as tested in a large observational data set provided by the PPMI cohort. We used a rigorous approach using models that considered the time‐varying nature of treatment initiation, thereby effectively removing some confounding that is present in simpler models.

Using these methods, we demonstrated that these data do not provide evidence that earlier treatment initiation leads to consistently worse motor symptoms, nonmotor symptoms, and functional disability. Motor scores seemed somewhat better for early starters (ie, those with longer treatment) in the first 4 years of follow‐up, but this benefit was only statistically significant at year 2 and for 1 of the models (IPTW) at year 4. These findings, made in a more real‐life environment with naturalistic medication prescription behavior, support previous findings from RCTs showing that earlier initiation of dopaminergic treatment does not accelerate clinical disease progression.^1^, ^2^, ^23^ We neither found strong evidence that earlier treatment start was associated with more side effects, that is levodopa‐induced dyskinesias or motor fluctuations, except for a small increase in dyskinesias at year 3. These findings should further alleviate concerns that early initiation of dopaminergic therapy leads to more severe side effects.

It is important to note that we aimed to estimate the effect of earlier treatment initiation, but from this analysis—which focused entirely on clinical outcomes, and not on biomarkers for underlying pathological disease progression—it is not possible to determine what mechanisms are at play. For example, patients who start on medication earlier may—because of their improved motor performance—have been able to better engage a more active lifestyle; an active lifestyle is also associated with lower MDS‐UPDRS motor scores.^24^, ^25^

We focused primarily on the effect of any type of PD medication, without prior discrimination between starting with levodopa, a dopamine agonist, or other drugs such as MAO‐B inhibitors. This enabled us to estimate the effect of longer exposure to increased cerebral dopaminergic stimulation, regardless of the drug that effectuates this. This approach is closest to clinical practice with its tremendous variety in how specific drugs are prescribed as primary treatment in early PD. Nevertheless, levodopa and dopamine agonists are intrinsically different, and the PD‐MED study already showed that levodopa might have a small beneficial effect on mobility relative to dopamine agonists or MAO‐B inhibitors.^6^ We therefore repeated our analysis including patients who started on levodopa only. However, the number of PPMI patients who started on levodopa only in the first 2 years of the disease was relatively small (n = 82). This made it difficult to accurately estimate the treatment effects of levodopa only and these results had, consequently, large confidence intervals and disagreements in the point estimates between the methods.

A strength of this study is that we used an observational data set with a relatively long follow‐up and broad inclusion criteria. Inclusion criteria in randomized controlled trials are often more restricted. For example, the LEAP study excluded patients if tremor was their most prominent complaint.^1^ Although the inclusion criteria in PPMI were broad, there might well be bias in this study population. For example, given the intensity of the follow‐up protocol, PPMI participants likely differ from a truly unselected outpatient population. Also, the enrolled PPMI participants were relatively young, and age unrepresentativeness is a well‐known problem in PD research.^26^ Ideally, the causal inference methods used here should also be applied to truly real‐life data sets, for example, derived from medical health care records or insurance companies, to mitigate this issue of selection bias. The large confidence intervals in our results also indicate that we would get more informative answers if the sample sizes had been substantially larger. It would therefore be interesting to repeat these analyses in larger cohorts, for example, the PD‐MED cohort with >1600 patients or the Critical Path Institute database, which merged multiple data sets.^2^, ^27^, ^28^

Our study was not without shortcomings. The outcome measures and the models we used come with limitations and assumptions that must be addressed to assess the relevance of our results. Although the MDS‐UPDRS III score as primary outcome is commonly used to quantify disease progression, its reliability to monitor individual changes in PD is low.^29^ More reliable instruments are needed to track individual disease progression, for example, by collecting rater‐independent continuous data using wearable sensors or using objective biomarkers. In addition, the sensitivity of MDS‐UPDRS Part III to the current LEDD of each patient makes it difficult to disentangle the disease‐modifying effects of early treatment initiation from the long‐duration response of LEDD on these measurements because earlier treatment initiation affects the LEDD. We were unable to assess the effect of treatment duration on quality of life, as there was no quality‐of‐life scale available within PPMI. To approximate the implications of earlier treatment start on daily life, we instead used the MDS‐UPDRS Parts I and II and the MSE‐ADL Scale.

The results of the causal models in this work rely on several important assumptions, including consistency, exchangeability, no model misspecification, and positivity.^14^ First, consistency relates to whether the treatment that was observed in the study is equivalent to the treatment that would be given if an intervention were to be implemented. In this case, it assumes that if an intervention on earlier treatment start were to be implemented, the choice of dosage and type of medication would be like those that were made in PPMI. We believe this is a reasonable assumption because PPMI included real‐world treatment decisions. Second, the exchangeability assumption relates to whether all variables that affect both outcome and choice of treatment at each time point have been accounted for. Although the variables included in the models offer a reasonable set of variables that reflect the disease state that will affect both treatment decision and subsequent outcomes, a more complete characterization could decrease potential bias from unmeasured confounders. Third, similar remarks hold for potential model misspecification introduced by the arguably simple functional forms of the models used here. The agreement between different models in most of our analyses gives confidence that the results are not only caused by model misspecification. Further development of models to predict treatment based on patient data^30^ and disease progression models^31^ will also help to increase the reliability of our conclusions. Finally, the positivity assumption in our case requires that even for disease states for which it is extremely likely (or unlikely) that therapy will be initiated, we assume there is always a small chance this does not happen (or does), as it does in daily practice.

In conclusion, this study showed the potential of using observational cohort data as an additional source of evidence for the effect of early treatment initiation in patients with de novo PD. Also, it underlines the importance of considering time‐varying confounding when interpreting data from cohort studies. With the increasing availability of large observational cohort studies, appropriate causal analyses can offer a valuable complement to the highly useful but hard to obtain and sometimes unrepresentative results from RCTs.

## Author Roles

(1) Research Project: A. Conception, B. Organization, C. Execution; (2) Statistical Analysis: A. Design, B. Execution, C. Review and Critique; (3) Manuscript: A. Writing of the First Draft, B. Review and Critique.

L.v.d.H.: 1A, 1B, 1C, 2C, 3A

L.E.: 1A, 2A, 2C, 3A, 3B

M.M.: 1A, 1B, 2C, 3B

A.S.: 1A, 2C, 3B

B.P.: 1A, 2C, 3B

T.H.: 1A, 1B, 2C, 3B

B.B.: 1A, 1B, 2C, 3B

J.K.: 1A, 1B, 1C, 2A, 2B, 3A

## Full financial disclosures for the previous 12 months

LvdH, LE, and JK received funding from a grant from the Netherlands Organization for Scientific Research (TOP Grant 91215076). LE received funding from Topconsortium voor Kennis en Innovatie, Life Sciences & Health, the Michael J. Fox Foundation, and Philips Research. MM received grants from the Ministry of Economic Affairs by means of the Personalized Parkinson Project Allowance made available by the Top Sector Life Sciences & Health to stimulate public–private partnerships, the Michael J Fox Foundation for Parkinson's Research, and the Horizon 2020 program. BP has nothing to disclose. AS was vice‐chair of the Advisory Committee Development and Implementation (Scientific Council), Dutch Cancer Society; was chair of the European Meeting of the Society for Medical Decision Making; received grants from the Dutch Cancer Society; and has received fees for speaking at the Netherlands Society for Geriatric Oncology and for training medical specialists in shared decision making at Maasstad Hospital, Haaglanden Medical Center. TH was partially financed by the Netherlands Organization for Scientific Research. BB currently serves as Editor‐in‐Chief for the *Journal of Parkinson*'*s Disease*; serves on the editorial board of *Practical Neurology and Digital Biomarkers*; has received honoraria from serving on the scientific advisory boards for Zambon, Biogen, UCB, and Walk with Path; has received fees for speaking at conferences from AbbVie, Zambon, Roche, GE Healthcare, and Bial; and has received research support from the Netherlands Organization for Scientific Research, the Michael J Fox Foundation, UCB, Abbvie, Zambon, the Stichting Parkinson Fonds, the Hersenstichting Nederland, the Parkinson's Foundation, Verily Life Sciences, Horizon 2020, the Topsector Life Sciences and Health, and the Parkinson Vereniging.

## Supporting information


**Appendix S1** Supporting Information
