# Supplementary material for: Estimating the Effect of Early Treatment Initiation in Parkinson's Disease Using Observational Data
Source: Mov Disord. 2020 Oct 27;36(2):407–14. doi: 10.1002/mds.28339 (PMC7984449; doi:10.1002/mds.28339)
Supplement: Supplementary file 1 — Appendix S1 Supporting Information [file MDS-36-407-s001.pdf]

Supplementary Material

Methodology

PPMI cohort

PPMI is an observational, longitudinal, multicenter study designed to establish a comprehensive set of clinical, imaging and biosample data that can be used to define biomarkers of PD progression. The PPMI cohort used for our study contained data from 423 recently diagnosed PD patients (15). Broad inclusion criteria consisted of 1) the presence of an asymmetric resting tremor, asymmetric bradykinesia or two of bradykinesia, resting tremor and rigidity; 2) diagnosis of PD for two years or less and not taking PD medications at screening; and 3) deficit consistent with PD on SPECT imaging. Patients were expected not to require PD medication within at least six months from the baseline measurement, however patients were retained in the study and continued treatment if this was required before six months of follow-up. All patients were assessed at the time of screening, at baseline (within 45 days of the screening visit), and every three to six months thereafter until a follow-up of 96 months. During each visit the investigator assessed a wide range of clinical measures and registered whether the patient used PD medication (yes/no) and, if so, which type of PD medication the patient used (multiple categories: levodopa, dopamine agonists, other, or any combination of those). Further information on the specific type of medication was described as free text in a medication log, from which the levodopa equivalent daily dose (LEDD) was derived

Statistical approach

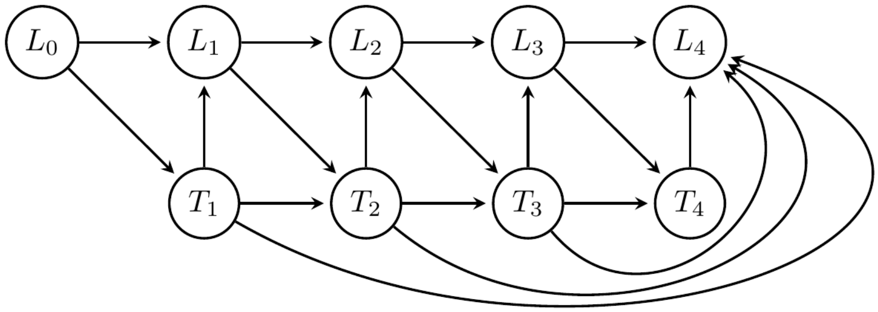

Figure 1S: Directed Acyclic Graph of an idealized version of our causal model. L represents the set of covariates and outcomes (MDS-UPDRS scores, MoCA, MSE-ADL, etc.) and T represents the treatment indicator. In addition, we assume there are background variables (age, sex, education, time since diagnosis) that may influence both treatment decisions and the covariates in L, and the full history of treatment decisions may influence the current disease state (not shown, except for the last node).

A simplified version of the underlying causal model we assume in this work is shown in Figure 1S. L in this figure denotes the time-varying confounders at each stage. These are formed by patient characteristics at each visit, and we assume they are adequately captured by the MDS-UPDRS part scores, MoCA and MSE-ADL in addition to the background characteristics of the patient: Age, Sex, Years of Education, disease duration and history of cardiovascular disease. An important insight from this model is that merely correcting for baseline measurements and characteristics is not enough to estimate the causal effect of intervening on the length of therapy in the first two years. Failing to properly adjust for the patient characteristics at each treatment decision could, for instance, improperly attribute longer therapy with bad outcomes, simply because the groups of patients that started medication therapy earlier, already had a more advanced state of the disease when the decision to start medication therapy was made. This confounding effect is known as time-varying confounding. The inverse probability of treatment weighting (IPTW) and parametric g-formula methods used in this work offer a way to adjust for this time-varying confounding, either by reweighting patients in such a way that in the reweighted sample the chance of receiving each treatment no longer depends on the covariates at each stage, or by modelling and simulating disease trajectories under all possible treatment assignments for each patient.

IPTW models

In IPTW, different weights are assigned to different patients to correct for the fact that the probability of starting treatment is not equal for each patient, like in a randomized experiment, but is influenced by the current disease state. Weighting patients differently is a way of equalizing these probabilities in the observational data to estimate what would have happened if the treatment initiation was properly randomized. To find the weights, at each visit we estimate the prior probability of starting treatment for those that have not started yet, which will be used to stabilize the weights. We then estimate the probability of starting treatment for those that have not started yet given the untransformed covariates (age, sex, years of education, disease duration, a binary indicator for history of cardiovascular disease, MDS-UPDRS part I, II and III scores, MSE-ADL scores and, if available, MoCA score) of only the previous visit, using a linear logistic regression model. Note that we do not pool the data across visits, which allows the model to be different for the different visits.

We divide the prior of the observed decision (started vs. not-started) by the probability of the observed decision given by this logistic regression model. By multiplying these weights for the different time-points, we get a final weight that is used for estimating the working marginal structural model. For censoring in the IPTW models, we use the same procedure as for the treatment weights, and multiply the censoring weight by the weight given by the treatment models to get an overall weight which we use in the working marginal structural model.

In the working marginal structural model (MSM), we use a linear model of the time on treatment. To avoid a mismatch between the continuous time treatment information and the discrete time confounding adjustments in our models, we define the time on treatment as the number of treatment periods the patient is recorded to be using PD medication,

multiplied by the length of the time periods (0.5 year). In other words, we define it to be $0.5 \sum_{i=1}^{4} t_i$, rather than the precise length of the treatment recorded in the study. In most analyses, in the working MSM, we adjust for the outcome measured at baseline (indicated here as “Baseline Adjusted”). In this supplement, we also report results when adjusting for all baseline variables, not just the particular outcome of interest (indicated by “Baseline Adjusted All”), and with and without adjustment for censoring using inverse probability of censoring weighting (indicated by “Censoring”). We also show results when adjusting for outcome at baseline and the Levodopa Equivalent Daily Dose of the patient at the time of the outcome measurement (indicated using “LEDD”). While this latter model may seem to be sensible to adjust for the long duration response of medication on the measurement, the causal interpretation of these results is not straightforward, since LEDD is likely influenced by the treatment decision whose effect we are trying to estimate. Finally, we use a linear regression model both for the continuous as well as for the dichotomous outcomes, which allows for both a uniform implementation of the models and presentation of the results. For the dichotomous outcomes, a logistic regression model would have been a sensible alternative choice.

Parametric g-formula models

When using the parametric g-formula, the goal was to estimate the effect of an intervention by constructing a model of disease progression over time, and we then used this model to simulate the effect of different interventions on the outcomes, starting from the observed values at baseline. We constructed models for the confounders and outcomes at each visit, based on data from the previous visit and the patient’s treatment status, using linear regression models. We allow for treatments earlier than the current visit to influence the prediction of the next observation. These links are not shown in the causal model in Figure 1S, but are reflected in the fitted model which contain the observed values of the covariates at the previous available time-point (and the baseline measurements, for the predictions at the final time-point) and all available previous treatment decisions. The variables that are included are treatment status, age, sex, years of education, disease duration, an indicator for the presence of cardiovascular disease, MDS-UPDRS part I, II and III scores, MSE-ADL and, for yearly measurements, MoCA score. To take the correlations between the time-varying covariates at each time point into account, we iteratively add the variables for the current time-point to the models, in the order just mentioned. For instance, the model to generate the current MDS-UPDRS part I score only contains variables from the previous visit, while the model for the current part II score includes the current part I score, and the model for the part III score includes the current part I and part II scores. Using these models, for each patient from the subpopulation of interest, for each of the 5 possible treatment decisions decisions (starting therapy at year 0, 0.5, 1, 1.5 or 2), we simulate 100 trajectories for the covariates and outcomes, which, in the analysis where we consider 5 options for the treatment length and the whole sample at baseline, gives (416*5*100=208000) trajectories. We use the same working marginal structural model on these simulated outcomes as used in the IPTW method to generate the final estimates.

Missing data

Since the efficiency of the estimators of some of our models improved by having access to complete observations at all time points under consideration, we imputed missing values. For the imputation of PD treatment status, we used the procedure described below in 'Missing treatment status'. For both the outcome measures and potential confounders, we imputed missing data as follows. If two consecutive measurements were missing we considered a patient as having dropped out of the study, and measurements at future time points were considered missing. If a single measurement was missing, we used the previous and next measurements to impute the missing values using a regression model estimated based on the observations of other patients that had no missing data at that time point.

Missing treatment status

If the information on treatment status was missing in a visit we instead use whether or not there was a positive LEDD in the medication log. For the use of levodopa specifically, we imputed as follows: if a patient used levodopa at the previous visit, we assumed the patient had started treatment and a positive levodopa status was imputed; if the patient had a negative levodopa status at the subsequent visit, we assumed the patient had not started levodopa and a negative levodopa status was imputed; if the levodopa status was negative at the previous visit but positive in the next visit, we assumed the patient also used levodopa at the current visit.

Supplementary Results

Overview of the presented analyses

Main analysis in bold, presented in manuscript.

Study sample	Intervention	Outcome measure	Model
• **Only starters**<br>• Only starters – extrapolated to whole PPMI population	• **Any PD medication**<br>• **Exclusively levodopa**	• **MDS-UPDRS III**<br>• **MDS-UPDRS I, II**<br>• **MoCA**<br>• **MSEADLG**<br>• **SCOPA-AUT**<br>• **Presence of dyskinesias & motor fluctuations**<br>• **QUIP (any & sum)**	• Naïve<br>• Baseline adjusted<br>• Baseline adjusted + censoring<br>• **Baseline adjusted + IPTW**<br>• **g-formula**

We also present the following sensitivity analyses, in which we investigate the effect of various modelling choices:

- Non-linearity of the effect
- Effect modification
- Investigation of the Treatment Probability Weights
- Sensitivity to alternative treatment model specifications
- Targeted Maximum Likelihood Estimation

Table 1S. Descriptives

Table 1S. Baseline characteristics for groups of different Parkinson Medication duration during the first two years since the start of the study. For continuous measures, we show the mean (standard deviation). For dichotomous measures, we show the proportion. Treatment Length indicates the number of years of PD medication treatment received by each group during the first 2 years since the start of the study, where Censored refers to the group of patients for whom no complete follow-up was available for the first two years. NA indicates number of missing values.

Treatment Length	0	0.5	1	1.5	2	Censored
n	58	37	54	180	31	56
Age (Years)	61.9 (10.6)	60.0 (9.0)	62.9 (9.9)	61.5 (9.5)	59.1 (10.9)	63.2 (8.2)
Gender (% Men)	0.60	0.62	0.69	0.67	0.71	0.66
Education (Years)	15.6 (2.7)	16.0 (2.1)	16.0 (2.5)	15.4 (3.1)	15.4 (3.0)	15.2 (3.6)
Disease Duration (Years)	0.8 (0.7)	0.6 (0.5)	0.6 (0.6)	0.5 (0.5)	0.4 (0.3)	0.5 (0.5)
MDS-UPDRS I	4.7 (3.3)	5.1 (4.0)	5.9 (4.2)	5.6 (3.9)	6.3 (5.3)	5.8 (4.1)
MDS-UPDRS II	3.7 (2.9)	4.6 (3.2)	5.7 (4.0)	6.7 (4.4)	6.3 (4.0)	6.4 (4.6)
MDS-UPDRS III	18.0 (7.8)	18.0 (7.7)	21.7 (9.3)	22.2 (8.6)	17.5 (8.7)	22.4 (9.7)
MoCA	27.1 (2.5)	26.9 (1.8)	26.9 (2.5)	27.1 (2.3)	27.0 (2.9)	27.4 (2.1)
MSE-ADL	95.4 (4.8)	93.5 (6.0)	93.1 (5.0)	92.7 (6.2)	92.1 (6.6)	92.8 (6.0)
SCOPA-AUT	8.2 (6.4)	8.9 (5.9)	9.2 (4.9)	10.1 (6.3), NA:1	8.8 (5.8)	9.6 (6.6)
QUIP-S (% any)	0.24	0.16	0.23	0.19	0.16	0.23
LEDD at 2 years	0.0 (0.0)	241.5 (232.6)	274.0 (172.4)	414.5 (324.9)	432.7 (244.0)	
LEDD at 3 years	125.1 (165.9), NA:6	326.3 (249.6), NA:2	337.4 (218.0), NA:2	525.1 (365.8), NA:12	564.0 (235.7), NA:3	
LEDD at 4 years	257.2 (244.9), NA:7	384.2 (275.2), NA:8	425.9 (240.9), NA:8	596.1 (379.9), NA:23	630.2 (294.4), NA:3	

Table 2S. Medication types

Table 2S. Distribution of types of medication use for patients in the different groups at year 2, according to questions answered by the investigator. Lv: levodopa; Ag: agonist.

Years Treatment	Missing	Lv	Ag	Other	Lv+Other	Lv+Ag	Ag+Other	Lv+Ag+Other
0.5	0.03	0.27	0.22	0.41	0.00	0.03	0.03	0.03
1.0	0.00	0.35	0.19	0.28	0.02	0.04	0.13	0.00
1.5	0.00	0.31	0.22	0.16	0.08	0.08	0.13	0.03
2.0	0.00	0.45	0.16	0.13	0.10	0.06	0.06	0.03

Figure 2S. MDS-UPDRS I, II, III, MoCA, MSEADL, SCOPA-AUT – Any PD medication – Only starters

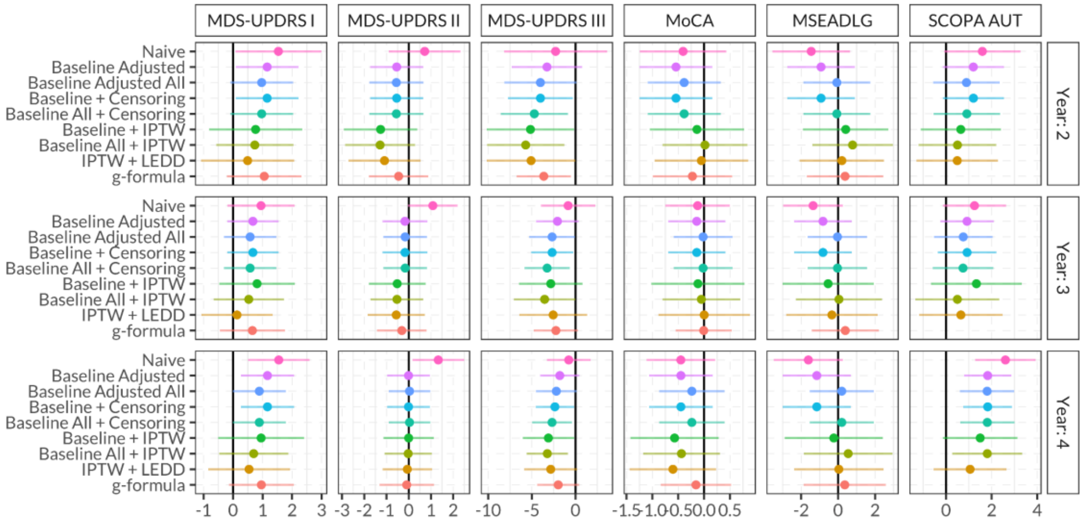

Figure 2S: Effect of a year of PD treatment (any medication) during the first two years of follow-up (estimated using only those patients who were using medication at the time of the outcome measurement). Effect on the score of different outcomes measured at year 2, 3 and 4.

Figure 3S. MDS-UPDRS I, II, III, MoCA, MSEADL, SCOPA-AUT – Any PD medication – Whole population

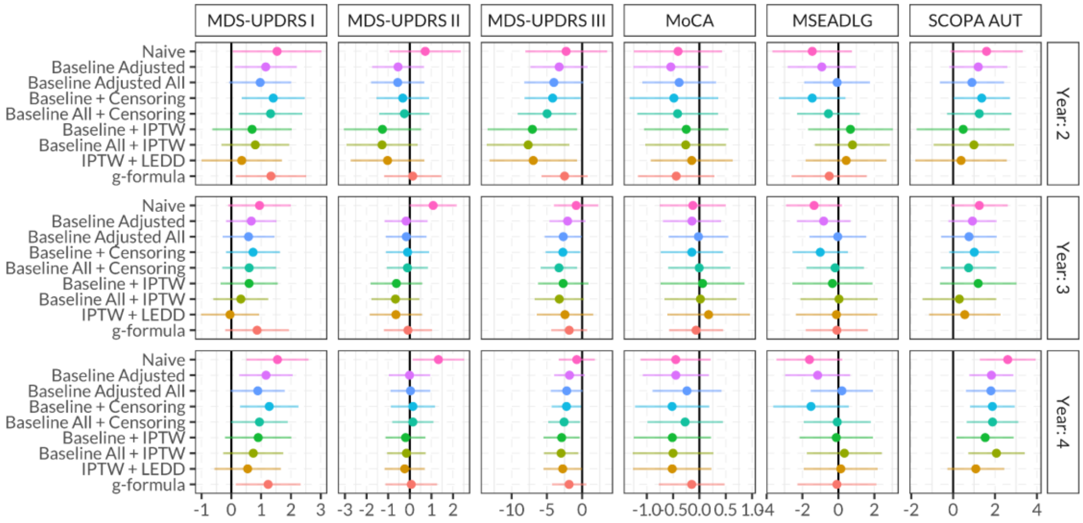

Figure 3S. Effect of a year of PD treatment (any medication) during the first two years of follow-up (extrapolated to the whole study population). Effect on the score of different outcomes measured at year 2, 3 and 4.

Figure 4S. MDS-UPDRS I, II, III, MoCA, MSEADL, SCOPA-AUT – Only levodopa – Only starters

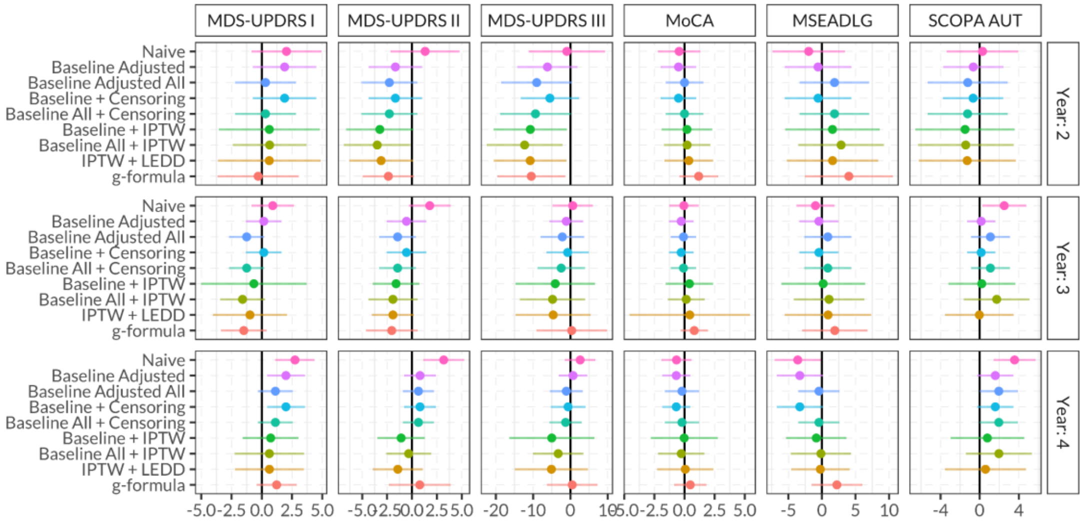

Figure 4S: Effect of a year of PD treatment (exclusively levodopa) during the first two years of follow-up (estimated using only those patients who used levodopa exclusively during the first two years). Effect on the score of different outcomes measured at year 2, 3 and 4.

Figure 5S. Dyskinesias, motor fluctuations, QUIP – Any PD medication – Only starters

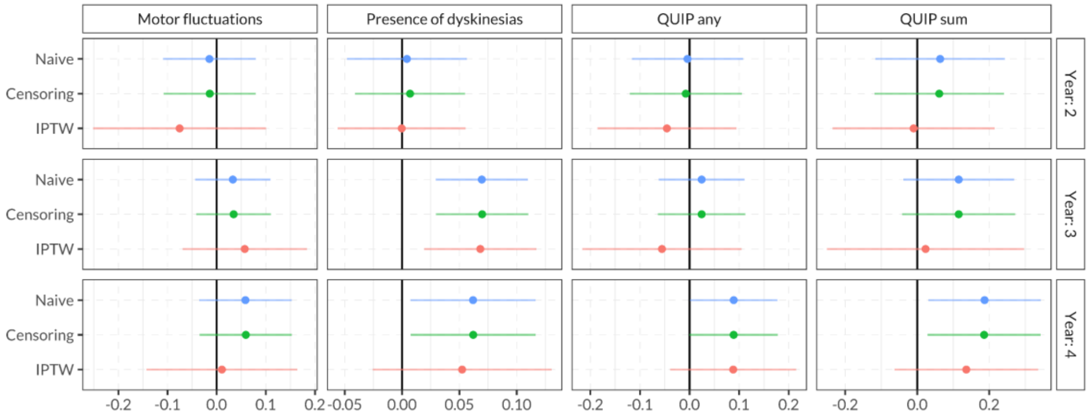

Figure 5S: Effect of a year of PD treatment (any medication) during the first two years of follow-up (estimated using only those patients who were using medication at the time of the outcome measurement). Effect on the score of different outcomes measured at year 2, 3 and 4. 'QUIP any' is a binary indicator of whether any of the behaviours in the QUIP questionnaire is present, while 'QUIP sum' refers to the total number of behaviours present (maximum of 8).

Figure 6S. Dyskinesias, motor fluctuations, QUIP – Any PD medication – Whole population

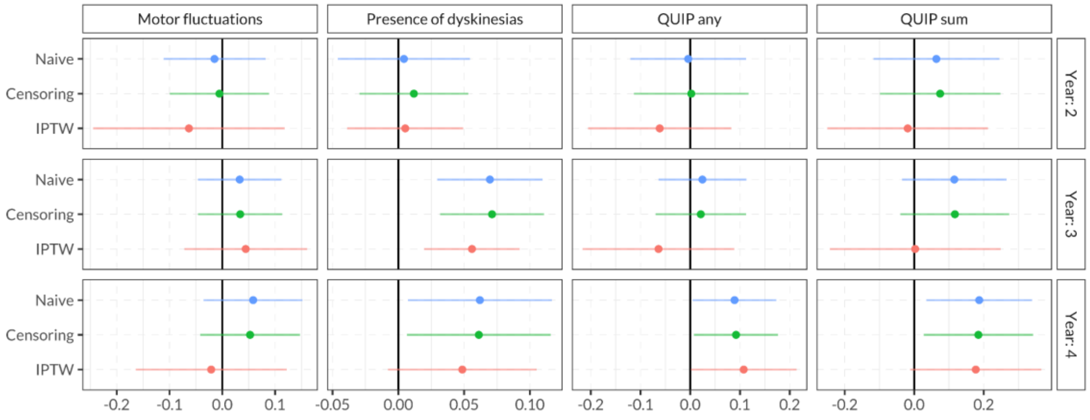

Figure 6S: Effect of a year of PD treatment (any medication) during the first two years of follow-up (extrapolated to the whole study population). Effect on the score of different outcomes measured at year 2, 3 and 4. 'QUIP any' is a binary indicator of whether any of the behaviours in the QUIP questionnaire is present, while 'QUIP sum' refers to the total number of behaviours present (maximum of 8).

Figure 7S. Dyskinesias, motor fluctuations, QUIP – Only levodopa – Only starters

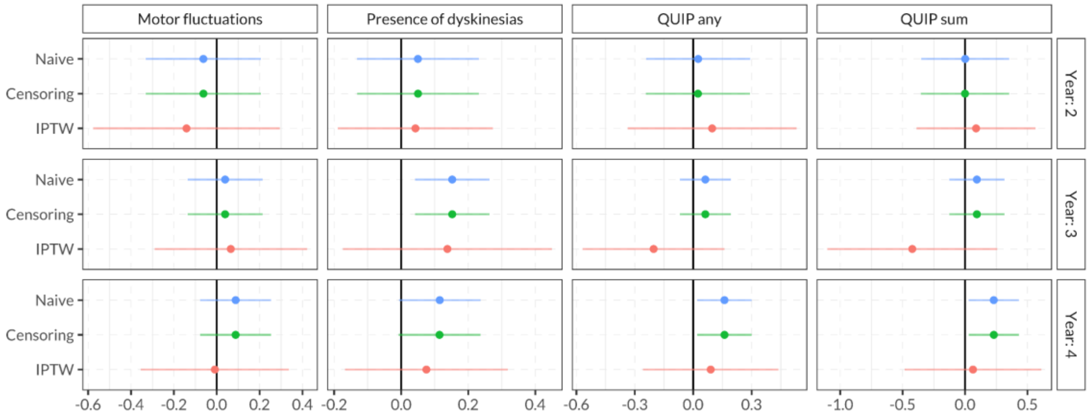

Figure 7S: Effect of a year of PD treatment (exclusively levodopa) during the first two years of follow-up (estimated using only those patients who used levodopa exclusively during the first two years). Effect on the score of different outcomes measured at year 2, 3 and 4. 'QUIP any' is a binary indicator of whether any of the behaviours in the QUIP questionnaire is present, while 'QUIP sum' refers to the total number of behaviours present (maximum of 8).

Non-linearity of the effect

In the results above we assumed in the marginal structural model that the effect of treatment was linear: adding half a year of treatment has the same effect regardless of whether one goes from 0 years of treatment to 0.5 year of treatment or from 1.5 years of treatment to 2 years of treatment. Here we estimate how the effect differs for different time points. "Treatment @ 2" refers to having treatment at year 2 (so in the final six months), Treatment 1.5 is between year 1 and 1.5, etc.

Figure 8S. Non-linearity of the effect – IPTW – Only starters

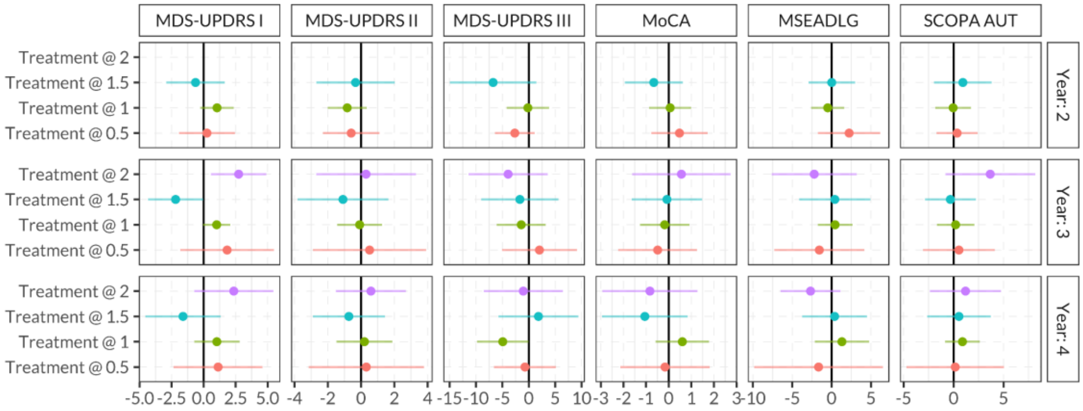

Figure 8S: Additive effect of treatment (any medication) being active at different time-points during the first two years for the IPTW model for only those patients who had started medication treatment at the time of the outcome measurement (hence, for the outcome at year 2, all included patients have active treatment at year 2). Effect on the score of different outcomes measured at year 2, 3 and 4.

Figure 9S. Non-linearity of the effect – g-formula – Only starters

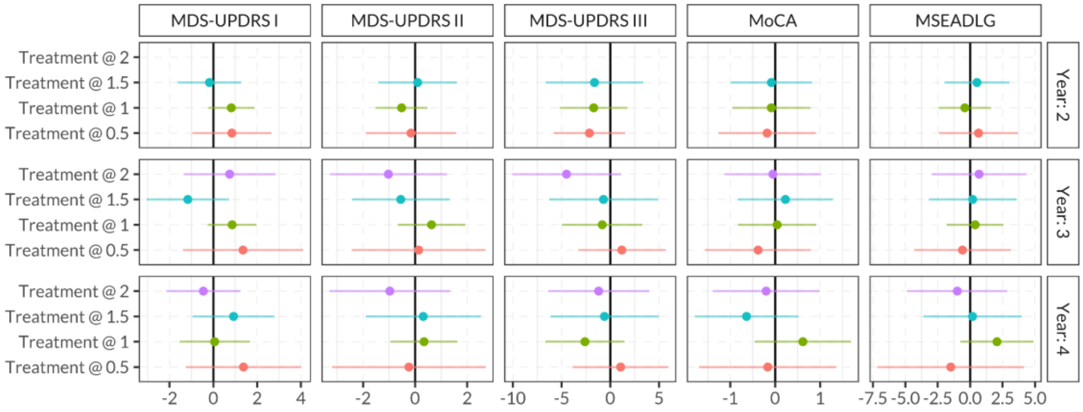

Figure 9S: Additive effect of treatment (any medication) being active at different time-points during the first two years for the g-formula model (estimated using those patients who had started medication treatment at the time of the outcome measurement). Effect on the score of different outcomes measured at year 2, 3 and 4.

Effect modification

To estimate the potential modification of the effect for different baseline characteristics of the patient, using the inverse probability of treatment weights for each outcome/year we estimate a working marginal structural model containing main effects and interaction terms of treatment length with age, sex, disease duration and three MDS-UPDRS subscores at baseline in a single model.

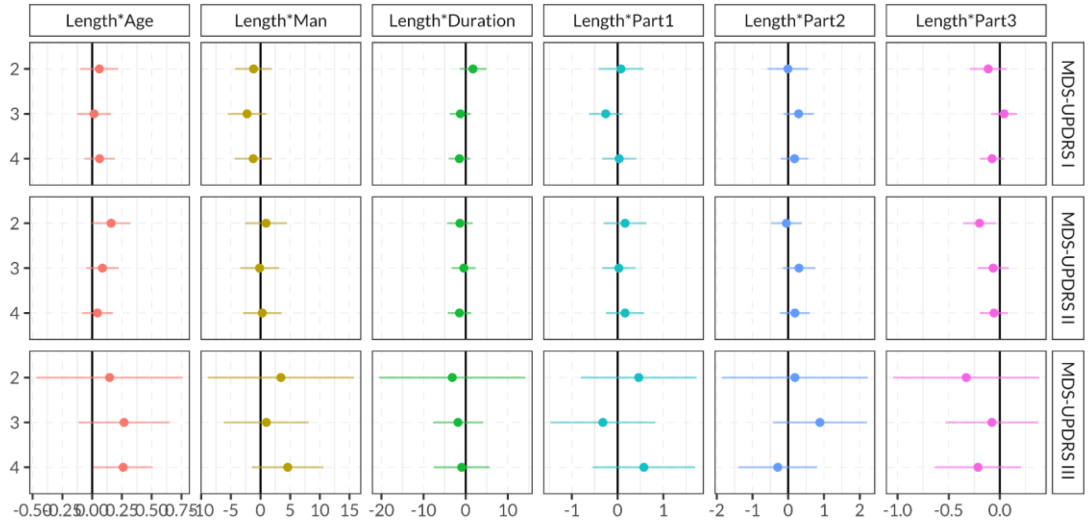

Figure 10S: Interaction effects in the IPTW Marginal Structural Model (main effects not shown), of the length of PD treatment (any PD medication) in the first two years of follow-up, on MDS UPDRS Part I, II and III, after 2, 3 and 4 years of follow-up, estimated using patients who had started medication therapy at the time of the outcome measurement.

Treatment Probability Weights

An important assumption for the causal effect of interest to be identified is the positivity assumption of the different treatments: for each treatment decision, both starting treatment and non-treatment have to have a positive probability of occuring, for all strata in the population of interest. Apart from identifiability issues, small treatment probabilities can also cause estimation issues in inverse probability weighting, since they lead to some observations receiving large weights. For our main analysis (MDS UPDRS part III, measured at year 2), we report the distribution of the estimated probability of the assigned treatment, the estimated probability of non-censoring, and the final stabilized weight used in our working marginal structural model. The bi-modal distribution in the propensity scores for treatment seems to be caused by the changing prior of starting treatment for the different time points. Because of the use of stabilized weights (where we multiply by the prior probability of the observed treatment assignment), the stabilized inverse probability weights do not show this strong bi-modality.

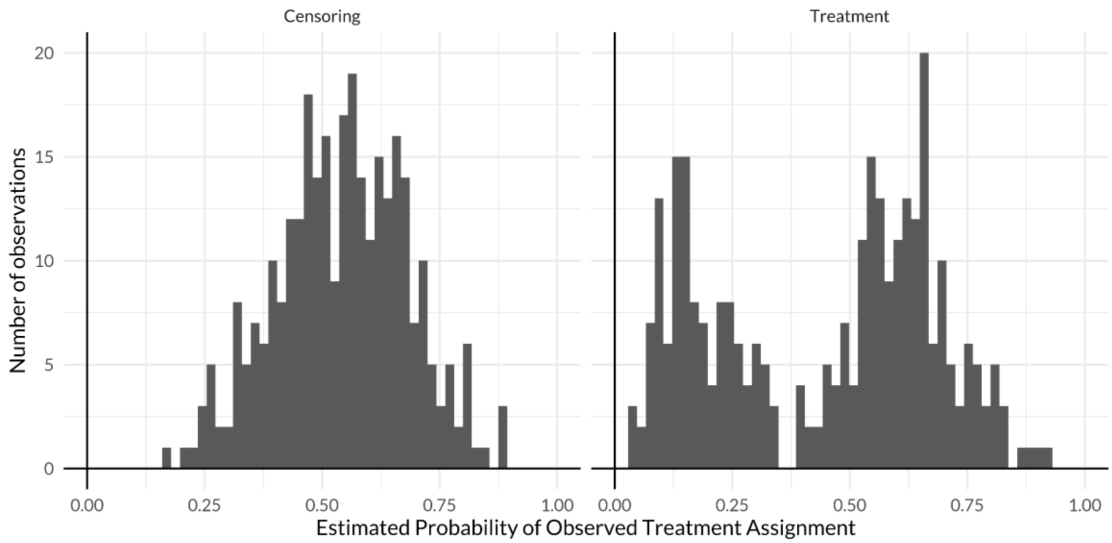

Figure 11S: Estimated probabilities of observed treatment and non-censoring for the IPTW model for MDS-UPDRS Part III 'OFF' scores at year 2 (estimated using only those patients who were using medication at the time of the outcome measurement).

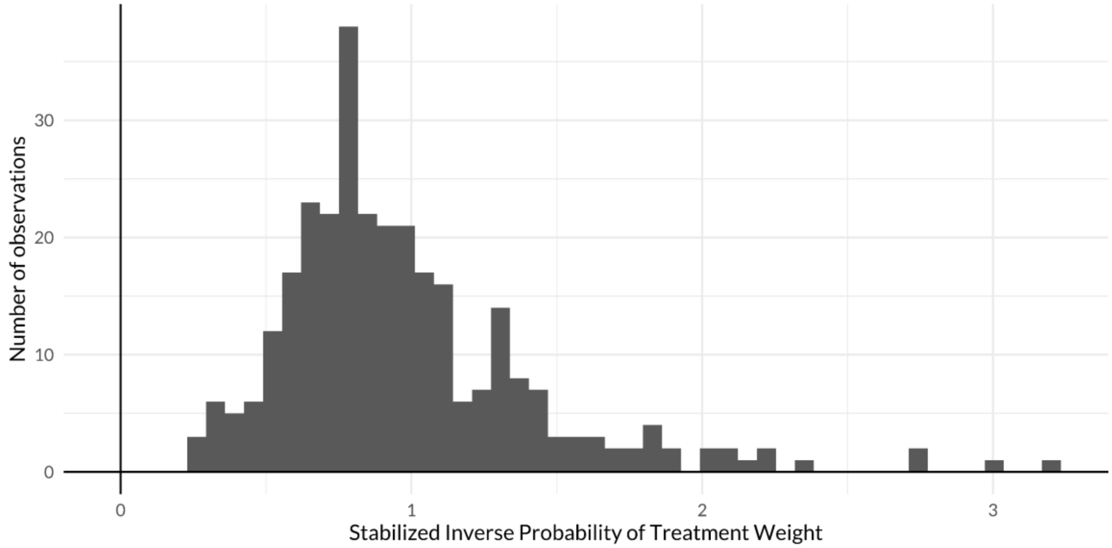

Figure 12S: Estimated stabilized inverse probability weights for the IPTW model for MDS-UPDRS Part III 'OFF' scores at year 2 (estimated using only those patients who were using medication at the time of the outcome measurement).

Sensitivity to alternative model specifications

Constructing models of complex disease and decision processes using limited data requires trade-offs in bias and variance of the model estimates. In the main analyses, we have focused on simple linear logistic and linear regression models, using a limited history of measurements (usually constrained to the previous measurements and constant patient

characteristics, rather than a longer measurement history). Here we investigate the effect of making different choices in the probability of treatment modelling.

We report the results of the IPTW estimates at year 2 when taking only the previous measurement into account (as in our main results, denoted here as “Markov” to reflect the Markov assumption), when taking full measurement history into account while still using linear logistic regression models (denoted as “Non-Markov”), as well as when using this full history for a non-parametric model, in this case a random forest classifier (denoted as “Non-parametric”). For the random forest classifier, we make use of R’s “randomForest” package, using the default settings, without cross-validation: 500 trees, a minimum node size 1, and the square root of the number of variables as the number of variables considered at each split. These defaults are also used in the “SuperLearner” implementation that we discuss in the next section.

The results show the increased variance caused by the larger number of features in the unregularized models. The non-parametric models lead to similar point estimates as our main analysis. We do not report the estimated confidence interval for these models, as these would likely be an underestimate of the true uncertainty.

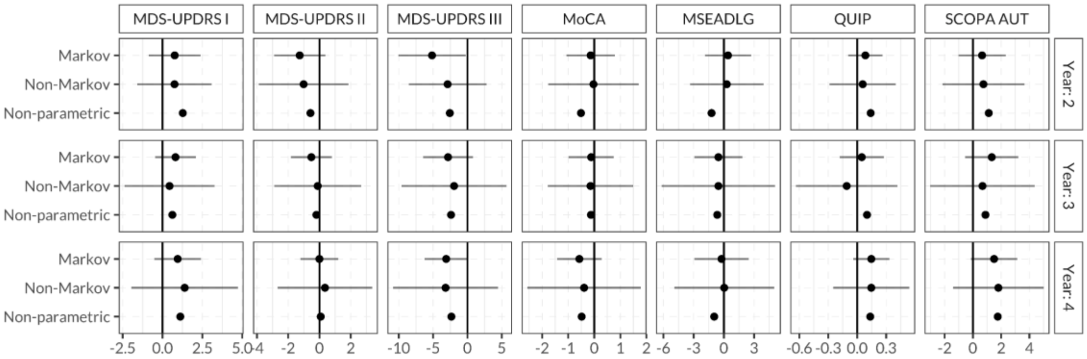

Figure 13S: Effect of a year of PD treatment (any medication) during the first two years of follow-up on outcomes at year 2 (estimated using only those patients who were using medication at the time of the outcome measurement) using the IPTW estimator and different propensity score models.

Targeted Maximum Likelihood Estimation

In our main analysis, we report on the performance of linear working MSMs with inverse probability weighting, as well as estimates using the parametric g-formula, and interpret the combination of their resulting estimates. An alternative is to combine these approaches into a single doubly robust estimate, with the guarantee that if either the probability of treatment model or the disease progression model (or both) is correctly specified, the combined estimator is a consistent estimator for the causal effect of interest. One elegant and efficient procedure to do this is targeted maximum likelihood estimation (van der Laan, M. J. (2010). Targeted maximum likelihood based causal inference: Part I. The international journal of biostatistics, 6(2).). We have attempted to apply this method to our

problem by using the ltmle R package (Lendle, S. D., Schwab, J., Petersen, M. L., & van der Laan, M. J. (2017). ltmle: an R package implementing targeted minimum loss-based estimation for longitudinal data. Journal of Statistical Software, 81(1), 1-21.). While this package covers a large number of modelling scenarios, it does not exactly map on to the problem setup we used in our main analyses, which makes it challenging to directly compare the results. Most importantly, the package uses logistic regression models on transformed outcomes (where outcomes are mapped to be in [0,1]) as the working MSM. While being an interesting modelling choice, the coefficients in these models do not directly correspond to the coefficients of the linear working MSM that we report in the main analyses.

The figure below shows the results of the estimates using the ltmle package for MDS-UPDRS Part III 'OFF' scores at the end of year 2, in a working MSM that includes the baseline MDS-UPDRS Part III scores. The propensity score and "Q" models include all previous measurements of the covariates (described as the "Non-Markov" setting in the previous section). We use both logistic regression for these models (results shown at the top of the figure) as well as a combination of logistic regression, Bayesian logistic regression and random forest models, that are combined using the SuperLearner package (see Van der Laan, M. J., Polley, E. C., & Hubbard, A. E. (2007). Super learner. Statistical applications in genetics and molecular biology, 6(1). and Polley, E., LeDell, E., Kennedy, C., Lendle, S., & van der Laan, M. (2019). Package 'SuperLearner'. https://CRAN.R-project.org/package=SuperLearner). For the SuperLearner model, Table 3S shows the contribution of the different models to the propensity score estimates at the different time points.

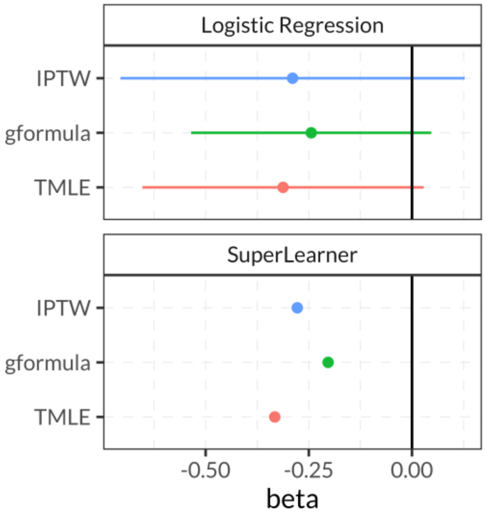

Figure 14S: Effect estimate of PD treatment (any medication) during the first two years of follow-up on outcomes at year 2 (estimated using only those patients who were using medication at the time of the outcome measurement) on MDS UPDRS Part III using the ltmle package for estimation. Note that the working MSM is a logistic regression model on the scaled outcomes, and the estimated beta is the parameter in this model, not the slope in the linear MSM used in the main analysis.

Table 3S: Weights of the different base models in the SuperLearner model for the propensity scores

	GLM	Random Forest	BayesGLM
Treatment @ 0.5y	0	0.43	0.57
Treatment @ 1y	0	0.92	0.08
Treatment @ 1.5y	0	1.00	0.00
Outcome Censored	0	0.95	0.05
